# Supplementary material for: Simple 3D-Printed Stirred Bioreactor Enhances Retinal Organoid Production Via Improved Oxygenation
Source: bioRxiv. 2025 Jun 20:2025.06.13.659558. Preprint. [Version 1] doi: 10.1101/2025.06.13.659558 (PMC12262459; doi:10.1101/2025.06.13.659558)
Supplement: 5 [file NIHPP2025.06.13.659558v1-supplement-5.pdf]

bioRxiv preprint doi: https://doi.org/10.1101/2025.06.13.659558; this version posted June 20, 2025. The copyright holder for this preprint (which was not certified by peer review) is the author/funder. This article is a US Government work. It is not subject to copyright under 17 USC 105 and is also made available for use under a CC0 license.

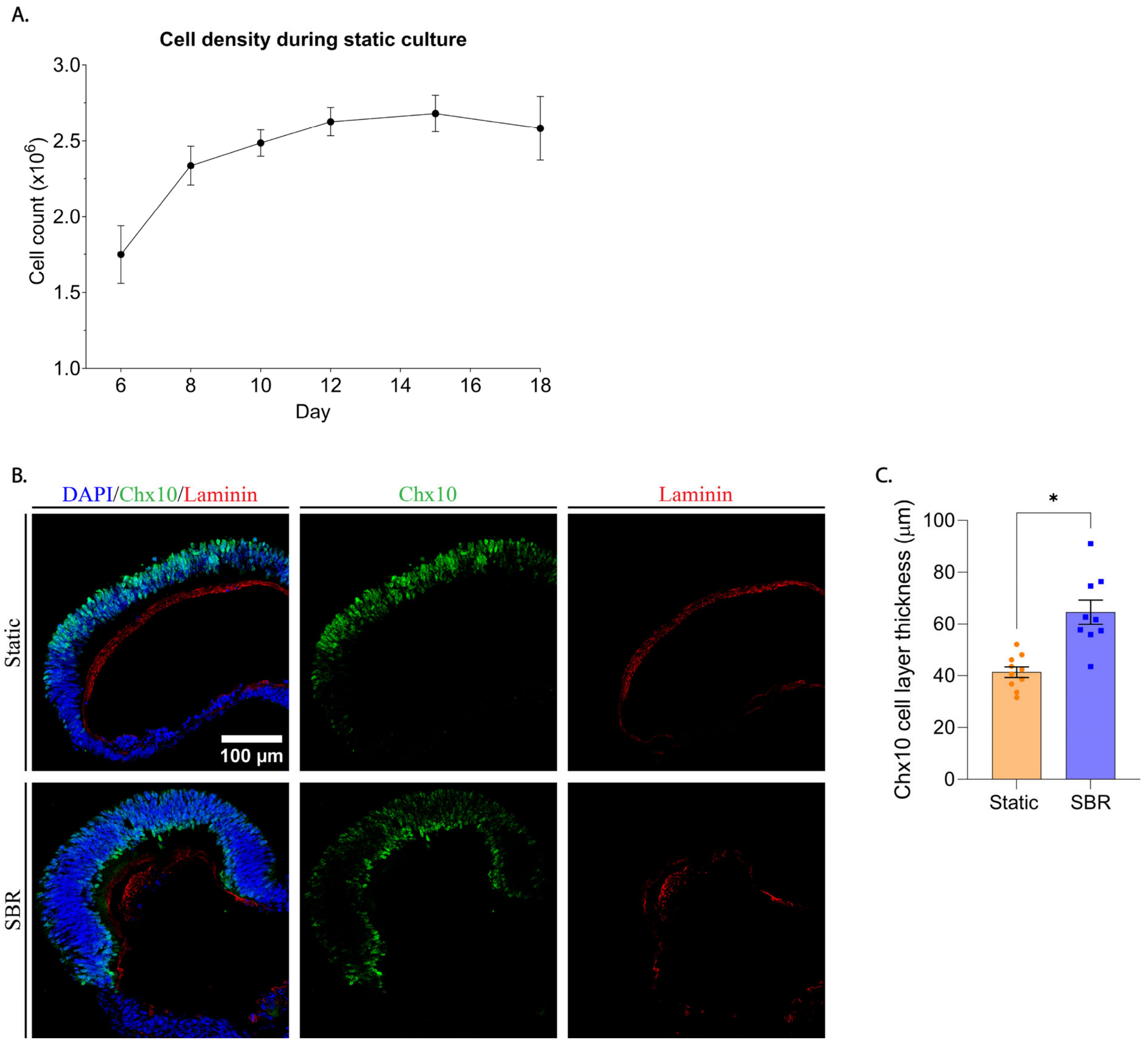

Supplementary Figure 1. Cell Density During Static Culture and Chx10 Cell Layer Thickness. ***A.*** *Analysis of cell density in static culture D6 to D18.* ***B.*** *Immunohistochemistry analysis of adherent OVs (hPSC1) using antibodies against markers for retinal progenitor cells or bipolar cells (CHX10, green), basement membrane (laminin, red). Nuclei were stained with 4',6-diamidino-2-phenylindole (DAPI, blue).* ***C.*** *Quantification of the CHX10^{+} cell layer thickness of hPSC1 ROs produced by static and SBR conditions. The bar charts summarized data from ten measurements. Presented as mean ± standard deviation*

Supplementary Table 1 (hPSC Lines)

Cell line	Lab ID	Origin	Sex	Publication
hPSC1	901A	Control individual	Male	37
hPSC2	8E	Control individual	Female	37
hPSC3	NEI001	Control individual	Female	38
hPSC4	904B	USH1C-Usher patient	Male	37
hPSC5	NRL-L75pfs	NRL Patient	Female	39

bioRxiv preprint doi: https://doi.org/10.1101/2025.06.13.659558; this version posted June 20, 2025. The copyright holder for this preprint (which was not certified by peer review) is the author/funder. This article is a US Government work. It is not subject to copyright under 17 USC 105 and is also made available for use under a CC0 license.

Supplementary Table 2 (Antibodies & Reagents)

Name/Target	Supplier	Catalog #	Host	Concentration
CHX10	Abcam	Ab16142	Sheep	1:500
ZO1	Thermo/Invitrogen	40-2200	Rabbit	1:500
DAPI	Thermo/Invitrogen	D1306	n/a	1:1000
**Hypoxyprobe-1 kit (hypoxic markers)**				
Pimonidazole	Hyproxyprobe	HP1-1000Kit	n/a	200 µM
MAb1	Hyproxyprobe	HP1-1000Kit	Rat	1:50
**In situ Cell Death Detection Kit (TUNEL assay, apoptotic markers)**				
TUNEL	Sigma (Roche)	12156792910	n/a	1:9 (Enzyme:Label solutions)

Supplementary Table 3 (OV yield & cross-sectional area analysis)

Cell line	Yield (mean)			Cross-sectional Area (μm^2, $n = 20$)		
	Static	SBR	*p*-value	Static	SBR	*p*-value
hPSC1 ($n = 5$)	204	387	0.013595	183584	261734	0.002664
hPSC2 ($n = 4$)	82	288	0.045679	192491	288162	0.009014
hPSC3 ($n = 3$)	292	488	0.031510	250530	412559	0.000491
hPSC4 ($n = 6$)	123	211	0.031099	265144	346462	0.003322
hPSC5 ($n = 3$)	33	177	0.000952	78622	170557	<0.000001
hPSC1-EB ($n = 3$)	78	193	0.005008	233503	414525	0.000014
hPSC2-EB ($n = 4$)	93	224	0.00698	176940	361093	0.000013

Supplementary Table 4 (Hypoxic and apoptotic IHC analysis)

Cell line	Hypoxia marker mean fluorescent intensity (A.U.)			Apoptosis marker TUNEL-positive cells to total nuclei (% ratio)		
	Static	SBR	*p*-value	Static	SBR	*p*-value
hPSC1	16819	6302	0.000073	18.69	5.348	0.001085
hPSC2	N/A	N/A		8.469	4.013	0.007352

bioRxiv preprint doi: https://doi.org/10.1101/2025.06.13.659558; this version posted June 20, 2025. The copyright holder for this preprint (which was not certified by peer review) is the author/funder. This article is a US Government work. It is not subject to copyright under 17 USC 105 and is also made available for use under a CC0 license.

Supplementary Table 5 (Shared DE Genes)

Shared Down		Shared Up	
**Gencode ID**	**Gene Symbol**	**Gencode ID**	**Gene Symbol**
ENSG00000131016	AKAP12	ENSG00000272808	AC015712.6
ENSG00000175040	CHST2	ENSG00000112319	EYA4
ENSG0000135111	TBX3	ENSG00000186007	LEMD1
ENSG00000255399	TBX5-AS1	ENSG00000198732	SMOC1
ENSG00000075035	WSCD2	ENSG00000018625	ATP1A2
ENSG00000211445	GPX3		
ENSG00000181408	UTS2R		
ENSG00000141052	MYOCD		
ENSG00000089225	TBX5		
ENSG00000151468	CCDC3		
